# KRIT1 Loss-Of-Function Associated with Cerebral Cavernous Malformation Disease Leads to Enhanced *S*-Glutathionylation of Distinct Structural and Regulatory Proteins

**DOI:** 10.3390/antiox8010027

**Published:** 2019-01-17

**Authors:** Laura Cianfruglia, Andrea Perrelli, Claudia Fornelli, Alessandro Magini, Stefania Gorbi, Anna Maria Salzano, Cinzia Antognelli, Francesca Retta, Valerio Benedetti, Paola Cassoni, Carla Emiliani, Giovanni Principato, Andrea Scaloni, Tatiana Armeni, Saverio Francesco Retta

**Affiliations:** 1 Department of Clinical Sciences (DISCO), Section of Biochemistry, Biology and Physics, Marche Polytechnic University, 60131 Ancona, Italy; l.cianfruglia@univpm.it (L.C.); principato@univpm.it (G.P.); 2 CCM Italia Research Network, National Coordination Center at the Department of Clinical and Biological Sciences, University of Turin, Orbassano, 10043 Torino, Italy; andrea.perrelli@unito.it (A.P.); claudia.fornelli@unito.it (C.F.); cinzia.antognelli@unipg.it (C.A.); francesca.retta@edu.unito.it (F.R.); valerio.benedetti@unito.it (V.B.); paola.cassoni@unito.it (P.C.); 3 Department of Clinical and Biological Sciences, University of Torino, Orbassano, 10043 Torino, Italy; 4 Department of Chemistry, Biology and Biotechnology, University of Perugia, 06123 Perugia, Italy; alessandro.magini@unipg.it (A.M.); carla.emiliani@unipg.it (C.E.); 5 Department of Life and Environmental Sciences (DISVA), Marche Polytechnic University, 60131 Ancona, Italy; s.gorbi@univpm.it; 6 Proteomics & Mass Spectrometry Laboratory, ISPAAM, National Research Council, 80147 Napoli, Italy; annamaria.salzano@cnr.it (A.M.S.); andrea.scaloni@ispaam.cnr.it (A.S.); 7 Department of Experimental Medicine, University of Perugia, 06132 Perugia, Italy; 8 Department of Medical Sciences, University of Torino, 10126 Torino, Italy

**Keywords:** cerebral cavernous malformations, KRIT1, oxidative stress, altered redox homeostasis and signaling, oxidative post-translational modifications, protein *S*-glutathionylation, redox proteomics, mass spectrometry

## Abstract

Loss-of-function mutations in the KRIT1 gene are associated with the pathogenesis of cerebral cavernous malformations (CCMs), a major cerebrovascular disease still awaiting therapies. Accumulating evidence demonstrates that KRIT1 plays an important role in major redox-sensitive mechanisms, including transcriptional pathways and autophagy, which play major roles in cellular homeostasis and defense against oxidative stress, raising the possibility that KRIT1 loss has pleiotropic effects on multiple redox-sensitive systems. Using previously established cellular models, we found that KRIT1 loss-of-function affects the glutathione (GSH) redox system, causing a significant decrease in total GSH levels and increase in oxidized glutathione disulfide (GSSG), with a consequent deficit in the GSH/GSSG redox ratio and GSH-mediated antioxidant capacity. Redox proteomic analyses showed that these effects are associated with increased *S*-glutathionylation of distinct proteins involved in adaptive responses to oxidative stress, including redox-sensitive chaperonins, metabolic enzymes, and cytoskeletal proteins, suggesting a novel molecular signature of KRIT1 loss-of-function. Besides providing further insights into the emerging pleiotropic functions of KRIT1, these findings point definitively to KRIT1 as a major player in redox biology, shedding new light on the mechanistic relationship between KRIT1 loss-of-function and enhanced cell sensitivity to oxidative stress, which may eventually lead to cellular dysfunctions and CCM disease pathogenesis.

## 1. Introduction

Cerebral cavernous malformation (CCM) is a major cerebrovascular disease of proven genetic origin (OMIM 116860), which is characterized by abnormally enlarged and leaky capillaries that predispose to seizures, neurological deficits, and intracerebral hemorrhage (ICH). It has been estimated to affect between 0.3% and 0.5% of the human population, and can occur either sporadically or as an inherited autosomal-dominant condition with incomplete penetrance and highly variable expressivity. The sporadic form often presents with a single lesion, while multiple lesions are generally observed in the familial form [1]. Three disease genes have been identified—KRIT1 (CCM1), CCM2, and PDCD10 (CCM3)—whose loss-of-function mutations are major determinants of disease pathogenesis. In particular, KRIT1 mutations have been associated with over 50% of all familial cases [2]. However, both clinical reports and accumulating evidence in animal models clearly demonstrate that homozygous loss of CCM genes is not fully sufficient to cause CCM lesion formation and disease progression, suggesting the necessary contribution of additional determinants, including microenvironmental stress events and interindividual variability in stress responses [3]. Consistently, the clinical behavior in individual patients, including the development of numerous and large lesions, and the risk of serious complications, such as ICH, remain highly unpredictable even among family members carrying the same mutation [1,4]. Indeed, although advances in the understanding of CCM disease pathophysiology have been significant, to date there are no direct therapeutic approaches for CCM disease besides the surgical removal of accessible lesions [5]. A complete, comprehensive understanding of the physiopathological functions of CCM proteins remains therefore a major research challenge for a more effective development of novel pharmacological strategies to limit disease progression and severity, and prevent *de novo* formation of CCM lesions in susceptible individuals.

Growing evidence demonstrates that CCM proteins are implicated in the modulation of redox-sensitive signaling pathways and mechanisms involved in adaptive responses to oxidative stress and inflammation, including major redox-sensitive transcriptional pathways and autophagy, thus contributing to the preservation of cellular integrity and homeostasis [3,6,7,8,9]. In particular, we previously demonstrated that KRIT1 plays a role in modulating master regulators of cellular redox homeostasis and responses to oxidative stress, including FoxO1, SOD2, and c-Jun, suggesting that altered redox signaling and oxidative stress contribute to CCM pathogenesis [7,8], and opening novel preventive and therapeutic perspectives [6]. Furthermore, we found that defective autophagy and enhanced production of reactive oxygen species (ROS) are common features of CCM gene loss-of-function and underlie major disease signatures, pointing to a novel unifying mechanistic scenario for CCM pathogenesis and treatment [9,10,11]. In addition, we identified genetic modifiers influencing disease severity, including polymorphisms of oxidative stress-related genes, suggesting that interindividual variability in susceptibility to oxidative stress may contribute to CCM disease pathogenesis and severity [4,12].

Here we addressed the possibility that KRIT1 loss-of-function exerts pleiotropic effects on distinct redox-sensitive mechanisms, including antioxidant defense mechanisms related to the tripeptide glutathione (L-γ-glutamyl-L-cysteinyl-glycine, GSH), the most abundant intracellular nonprotein thiol and a key modulator of redox-sensitive cellular functions. Indeed, the glutathione redox buffer system, including an optimal ratio of reduced to oxidized glutathione (GSH/GSSG), plays an essential role in maintenance of intracellular redox homeostasis, which is in turn critical for proper function of fundamental proteins and cellular processes, as well as for cellular defense against oxidative damage. Conversely, a decline in the GSH/GSSG ratio is considered a marker of oxidative stress, and has been associated with increased protein *S*-glutathionylation (PSSG), a highly conserved oxidative post-translational modification (OPTM) consisting of the reversible formation of mixed disulfides between the thiol of GSH and a thiol group of a target protein [13,14]. In turn, PSSG has now emerged as an important mechanism for dynamic, post-translational regulation of a variety of structural, metabolic, and regulatory proteins, providing protection of proteins against irreversible oxidation of critical cysteine residues, and influencing transduction of redox signals involved in multiple physiopathological processes, including cellular responses to oxidative stress in the cardiovascular system [15,16,17,18].

The outcomes of our experiments demonstrated that KRIT1 deficiency affects the GSH redox buffer system, causing downregulation of the GSH/GSSG ratio and enhanced *S*-glutathionylation of distinct proteins involved in adaptive responses to oxidative stress, including redox-sensitive chaperonins, metabolic enzymes, and cytoskeletal proteins, thus uncovering a novel molecular signature of KRIT1 loss-of-function with potential physiopathological and therapeutic implications.

## 2. Materials and Methods

### 2.1. Reagents

Cell culture reagents and ECL LiteAblot were obtained from Euroclone (Milan, Italy). Chemical reagents and secondary antibodies were from Sigma-Aldrich (St. Louis, MO, USA). Carboxy-H2DCFDA (C400) was obtained from Invitrogen (Carlsbad, CA, USA). Mouse monoclonal anti-glutathione was obtained from Santa Cruz Biotechnology (Heidelberg, Germany). Immobiline™DryStrips pH 3–10 NL were supplied by GE Healthcare (GE Healtcare, Buckinghamshire, UK). Bradford reagent and polyvinylidene difluoride (PVDF) membranes were obtained from Bio-Rad (Hercules, CA, USA).

### 2.2. Cell Culture and Treatment

KRIT1^−/−^ (K^−/−^) and KRIT1^+/+^ (K^9/6^) mouse embryonic fibroblast (MEF) isogenic cell lines were previously described [7]. Specifically, K^9/6^ cells were derived from K^−/−^ MEFs by lentiviral re-expression of KRIT1 in order to obtain KRIT1-null and KRIT1-expressing MEF cells with uniform genetic backgrounds to be used for comparative molecular and cellular biology studies [7]. Cells were cultured in DMEM containing 10% *v*/*v* heat-inactivated fetal bovine serum (FBS), 2 mM glutamine, 10 U/mL penicillin, and 10 µg/mL streptomycin, at 37 °C and 5% CO_2_. Human brain microvascular endothelial cells (hBMEC) were purchased from ScienCell Research Laboratory (Carlsbad, CA, USA) and cultured in EGM-2MV medium (Lonza, Basel, Switzerland) at 37 °C and 5% CO_2_ on cell culture dishes coated with rat tail collagen type-I (BD Biosciences, San Jose, CA, USA). Cell viability was estimated by examining the ability to exclude 0.1% Trypan Blue in 0.9% NaCl. For GSH treatment, cells were subcultured in 6-well plates at a concentration of 3 × 10^5^ cells/well, and then incubated with 5 mM GSH-encapsulated liposomes for 4 h [19]. Cell treatment with antioxidant compounds, including the mitochondria-permeable Tiron (4,5-dihydroxy-1,3-benzenedisulfonic acid disodium salt monohydrate, Sigma-Aldrich, Milan, Italy) (5 mM for 24 h), was performed as previously described [20]. The concentrations and timing of GSH and Tiron used for rescue experiments were based on preliminary dose-response and time course experiments performed in previous works [19], which showed an optimal efficacy of the indicated concentration and time of treatment.

### 2.3. Cell Transfection and siRNA-Mediated Gene Silencing

KRIT1 knockdown in hBMEC cells was performed by RNA interference (RNAi), as previously described [9,20]. Briefly, cells cultured to 50–70% confluence in a 6­well plate with 2.5 mL of complete EGM-2MV medium were transfected with either a mix of four predesigned iBONi small interfering RNAs (siRNAs) targeting KRIT1 (D-00101-Plus, Ribbox GmbH, Radebeul, Germany), or a negative control iBONi siRNA (K-00301-0005-N3, Ribbox GmbH). The transfection complex was prepared by mixing 25 nM iBONi siRNAs and 1:166 HiPerFect reagent (Qiagen, Milan, Italy) in serum­free medium followed by incubation at room temperature for 15 min, and then was added dropwise onto the cells. To obtain higher levels of KRIT1 knockdown, the siRNA transfection procedure was repeated after 48 h. Cells were then were plated into assay dishes and subjected to experimental conditions within 48–72 h. The efficiency of KRIT1 silencing was monitored by qRT-PCR and Western blotting analysis.

### 2.4. Preparation of Liposomes

Liposomes were prepared using egg phophatidylcholine (PC) (Sigma-Aldrich) and the extrusion technique with a small extruder apparatus (Avanti Polar Lipids Inc., Alabaster, AL, USA), as previously described [21] using a small extruder apparatus (Avanti Polar Lipids Inc., Alabaster, AL, USA). Briefly, before extrusion, a lipid film was prepared in a glass vial by dissolving the lipid-soluble stock solution in chloromethane, and then evaporating the solvent by a stream of nitrogen. The lipid film was then hydrated with 150 mM GSH, and solubilized large multilamellar vesicles (LUVs) were extruded at 50 °C through polycarbonate membranes with a nominal pore diameter of 100 nm (Avanti Polar Lipids). For cell treatments, encapsulated liposomes containing 150 mM GSH were diluted in serum-free medium to reach a final concentration of 5 mM [19].

### 2.5. Preparation of Cellular Extracts

Cultured cells were washed twice with 0.9% NaCl at 4 °C. Protein extracts for Sodium Dodecyl Sulphate-PolyAcrylamide Gel Electrophoresis (SDS-PAGE) and immunoblotting were obtained by quickly treating cells with 40 mM N-ethylmaleimide for 10 min, at 4 °C. This treatment avoided the formation of protein mixed disulfide with glutathione (PSSG) or disulfide scrambling phenomena due to sample manipulation. Cells were then centrifuged at 500× *g* for 5 min, at 4 °C, and finally lysed with 10 mM sodium phosphate, pH 6.0, containing 0.5% *v*/*v* Nonidet P40, at 4 °C. After 30 min incubation on ice, cell lysates were centrifuged at 13,000× *g* for 15 min, at 4 °C. Supernatants were then recovered and total protein concentration was determined by the Bradford protein assay.

For antioxidant activity assays, cells were resuspended in phosphate buffered saline (PBS) containing aprotinin (1 μg/mL), freeze-thawed in liquid nitrogen three times, and centrifuged at 12,000× *g* for 10 min, at 4 °C. In this case, detergents were not used, as they are known to interfere with total oxyradical scavenging capacity (TOSC) determinations [22]. Cellular antioxidant activity was analyzed in cell extracts by measuring the levels of TOSC toward hydroxyl radicals, and the activities of the main antioxidant enzymes (CAT, GST, GR, and Se-dependent and Se-independent GPX).

### 2.6. Measurement of Cellular Levels of Reactive Oxidative Species

Assessment of cellular levels of reactive oxidative species, including levels of general reactive oxygen species (ROS) and reactive nitrogen species (RNS), was performed by flow cytometry using the fluorogenic probe 6-carboxy-2′,7′-dichlorodihydrofluorescein diacetate (carboxy-H_2_DCFDA) (Invitrogen), as a follow-up analysis of previously published studies [7,8,9,20,23,24]. Specifically, the cell-permeant carboxy-H_2_DCFDA passively diffuses into cells where it is converted to dichlorodihydrofluorescein (H_2_DCF) after cleavage of the acetate groups by intracellular esterases. The resulting nonfluorescent H_2_DCF is then trapped intracellularly due to its hydrophilicity and oxidized by various ROS/RNS to form the highly fluorescent 2′,7′-dichlorofluorescein (DCF), thus serving as an effective indicator of generalized cellular oxidative stress. Briefly, cells were trypsinized, washed twice with PBS, suspended in PBS containing 10 µM carboxy-H_2_DCFDA (C400), and incubated for 30 min, at 37 °C. Samples were then washed twice in PBS and labeled with 10 µg/mL propidium iodine (PI) to remove false negative results due to cells with compromised plasma membrane. Finally, samples were immediately analyzed for fluorescence intensity with an EPICS XL flow cytometer (Beckman Coulter, Brea, CA, USA) using a 488-nm excitation beam and a 538-nm band-pass filter. Median fluorescence intensity was quantified by FCS Express (DeNovo Software, Glendale, CA, USA) analysis of the recorded histograms. More specific and consistent measurements of intracellular ROS levels in the same cellular models were performed previously using the superoxide (O_2_^•−^) indicator MitoSOX Red [7], the H_2_O_2_ sensor pHyPer-dMito (mt-HyPer probe) (Vinci-Biochem, Vinci, FI, Italy) [9], and the Amplex^®^ Red Hydrogen Peroxide/Peroxidase Assay Kit (A22188, Invitrogen, Milan, Italy) [23].

### 2.7. Total Oxyradical Scavenging Capacity Assay

The total antioxidant capacity (TAC) of cell extracts was evaluated using the total oxyradical scavenging capacity (TOSC) assay, which measures the capacity of cellular antioxidants to inhibit the oxidation of α-keto-γ-methiolbutyric acid (KMBA) to ethylene gas in the presence of different forms of oxyradicals, artificially generated at a constant rate. Reactions were performed on 50 µL of cell extracts and 2 mM KMBA; hydroxyl radicals were generated from the Fenton reaction of iron-EDTA (1.8 µM Fe^3+^, 3.6 µM EDTA) plus ascorbate (180 µM) in potassium phosphate buffer (100 mM, pH 7.4). TOSC values were quantified using the equation TOSC = 100 − (SA/CA × 100) and referred to the protein concentration of each sample, as previously described [25,26]. Specifically, SA and CA represent the integrated area calculated under the least squares kinetic curve produced during the reaction for the sample and the control, respectively.

### 2.8. Quantitative Determination of Glutathione and Glutathione Disulfide Levels

Total glutathione (GSH+GSSG) and oxidized glutathione (GSSG) were measured spectrophotometrically (at 412 nm) using the glutathione reductase (GR) recycling assay in the presence of 5,5′-dithiobis(2-nitrobenzoic acid) (DTNB), with a calibration line based upon known concentrations of GSH and GSSG [27]. To prevent GSH artificial oxidation during sample processing, cells were washed twice (1 min each) at room temperature with PBS containing 5 mM N-ethylmaleimide (NEM) (Sigma Aldrich, Milan, Italy) immediately after culture medium removal, according to an optimized protocol for the reliable measurement of GSH, GSSG, and PSSG in cell cultures [28]. Cells were then trypsinized, washed twice in cold PBS containing 5 mM NEM, and quickly centrifuged. For total GSH/GSSG determination, the pellet was resuspended with 1% sulfosalicylic acid, vortexed, and incubated 30 min at 4 °C. Samples were then centrifuged at 2300× *g* for 2 min, and the supernatant was separated in two aliquots: one was maintained at 4 °C for GSH quantification, and the other was treated with 2-vinilpiridin (Cf = 5%) and 20% *v*/*v* triethanolamine (Cf = 1%) to mask the GSH present in the extract and prevent its measurement. Finally, the pellet was resuspended with 1 M NaOH for recovery and quantification of proteins [26].

### 2.9. Enzymatic Activity Assays

Glutathione reductase (GR) activity was analyzed by the method described by Carlberg and Mannervik [29], which measures the decrease in absorbance at 340 nm due to NADPH oxidation during GSSG reduction. The assay was performed in 100 mM sodium phosphate (pH 7.0), 0.1 mM NADPH, and 1 mM GSSG; GR activity was calculated using an extinction coefficient (ε) for NADPH of 6.22 mM^−1^ × cm^−1^ and expressed as µmol of NADP^+^ per min per mg of proteins.

Glutathione-S-transferase (GST) activity was analyzed using 1-chloro-2,4-dinitrobenzene (CDNB) as substrate and measuring the absorbance of resulting products at 340 nm, according the method of Habig and colleagues [30]. Specifically, this colorimetric assay relies on the reaction between GSH and CDNB that is catalyzed by a broad range of GST isozymes, including alpha, mu, pi, and other GST isoforms. The assay was performed in 100 mM sodium phosphate (pH 6.5), 1 mM CDNB, and 1 mM GSH. GST activity (defined as the amount of enzyme producing 1 µmol of CDNB-GSH conjugate/min under the conditions of the assay) was calculated using an extinction coefficient (ε) for CDNB of 9.6 mM^−1^ × cm^−1^, and expressed as µmol of CDNB-GSH conjugates per min per mg of proteins [26].

Glutathione peroxidase (GPX) activity was assayed in a coupled enzyme system, where NADPH is consumed by glutathione reductase to convert the formed GSSG to its reduced form (GSH). The decrease of absorbance was monitored at 340 nm (ε = 6.22 mM^−1^ × cm^−1^) using 0.8 mM cumene hydroperoxide as substrate for the sum of Se-dependent and Se-independent enzyme forms, in 100 mM potassium phosphate, pH 7.5, 1 mM EDTA, 1 mM dithiothreitol, 2 mM GSH, 0.24 mM NADPH, and 1 unit of GR.

Catalase (CAT) activity was determined using 12 mM hydrogen peroxide (H_2_O_2_) in 100 mM potassium phosphate, pH 7.0, as substrate, and measuring the decrease in absorbance at 240 nm (ε = 0.04 mM^−1^ × cm^−1^) due to the consumption of H_2_O_2_.

### 2.10. Western Blotting Analysis

Cell extracts (30 µg total proteins) were analyzed by 12% SDS-PAGE. Analysis was carried out in nonreducing conditions to avoid reduction of PSSG derivatives. Proteins were then transferred onto a PVDF membrane, which was then blocked with 5% nonfat dry milk in Tris-buffered saline containing 0.1% Tween 20 (TBST), at 25 °C, for 1 h. Protein *S*-glutathionylation levels were analyzed by Western blotting with a specific mouse monoclonal primary antibody that recognizes GSH-protein complexes (ViroGen, Watertown, MA, USA), and a goat anti-mouse HRP-linked secondary antibody, and detected by chemiluminescence, as previously described [19,26]. KRIT1 silencing in endothelial cells was assessed using a specific rabbit mAb (Ab196025, Abcam, Cambridge, UK). As internal control for protein loading, membranes were reprobed with antibodies for housekeeping proteins, including β-actin (mAb C4, Santa Cruz Biotechnology, Heidelberg, Germany), as previously described [20]. 

### 2.11. Immunoprecipitation

Immunoprecipitation (IP) was performed using Dynabeads Protein G Immunoprecipitation Kit (Invitrogen, Life Technologies, Milan, Italy) according to the manufacturer’s instructions. Briefly, cells were lysed in ice-cold RIPA lysis buffer added with a cocktail of protease inhibitors. The lysate was incubated with Dynabeads protein G conjugated with a mouse anti-Hsp60 mAb (H-1: sc-13115, Santa Cruz Biotechnology). Immune complexes were analyzed by Western blot using either anti-GSH or anti-Hsp60 antibodies as described above.

### 2.12. Two-Dimensional Polyacrylamide Gel Electrophoresis (2D PAGE) Analysis

Cells were harvested and resuspended in 8 M urea, 40 mM Tris and 2% CHAPS to allow the recovery of total protein extracts. Protein content was quantified by the Bradford assay. Isoelectrofocusing (IEF) (first dimension) was performed using a 7 cm Immobiline™DryStrip NL (GE Healthcare, Milan, Italy), pH 3–10. Each strip was actively rehydrated in the presence of proteins (200 µg) within an IPG-Phor system (Amersham Pharmacia, Macclesfield, UK) at 30 V, for 12 h. After rehydration, IEF runs were carried out at 20 °C, based on a current limit of 50 µA/IPG-strip: 300 V for 1 h; 4000 V for 3.75 h (gradient); and 4000 V until 15,000 V/h was reached in total. After IEF, IPG-strips were equilibrated in a buffer containing 50 mM Tris-HCl, pH 8.8, 6 M urea, 30% *v*/*v* glycerol, 2% *w*/*v* SDS, for 30 min. SDS-PAGE (second dimension) was performed at 90 V for 130 min using 10% acrylamide/bis-acrylamide gels. First and second dimensions were carried out in nonreducing conditions to avoid reduction of PSSG derivatives. Gels were then blotted onto PVDF membranes, and then Western blotting with a mouse monoclonal anti-GSH antibody was performed to analyze protein *S*-glutathionylation levels, as previously described [19,26]. Parallel 2-DE gels were directly stained with colloidal Coomassie Blue [31]. Digitalized gel images of Western blotting membranes and colloidal Coomassie-stained gels were acquired using an Image Scanner II (GE Healthcare, Bronx, NY, USA) apparatus and analyzed with the Image Master Platinum 6.0 software (GE Healthcare) for spot matching and relative quantization. Protein spots corresponding to immunoreactive signals were manually excised from 2D-gels and further subjected to mass spectrometric analysis.

### 2.13. Identification of S-Glutathionylated Proteins by Mass Spectrometry Analysis

Gel spots were triturated, washed with water, S-alkylated, and digested with sequencing-grade trypsin (Sigma, St. Louis, MO, USA) [32]. Peptide digests were desalted using μZip-TipC18 (Millipore, Bedford, MA, USA) and 50% *v*/*v* acetonitrile, 5% *v*/*v* formic acid as eluent, and directly analyzed by nanoliquid chromatography-electrospray ionization-linear ion trap-tandem mass spectrometry (nLC-ESI-LIT-MS/MS). Experiments were performed with a LTQ-XL mass spectrometer (Thermo Fischer Scientific, Waltham, MA, USA) equipped with a Proxeon nanospray source connected to an Easy-nanoLC (Proxeon, Odense, Denmark) [33]. Peptide mixtures were separated on an Easy C18 column (100 × 0.075 mm, 3 μm) (Thermo Fischer Scientific, Waltham, MA, USA). Mobile phases were 0.1% *v*/*v* formic acid (solvent A) and 0.1% *v*/*v* formic acid in acetonitrile (solvent B), running at a total flow rate of 300 nL/min. Solvent B was ramped from 5% to 35% over 15 min and from 35% to 95% over 10 min. Spectra acquisition in the range 400–2000 *m*/*z* was controlled by data-dependent product ion scanning (Top3-MS2 ), setting mass isolation window and collision energy to 3% and 35%, respectively, and enabling dynamic exclusion for 60 s with repeat count 2.

Raw data from nLC-ESI-LIT-MS/MS analysis were subjected to a MASCOT search (v.2.3.02, Matrix Science, London, UK) against a UniProtKB Mus musculus protein sequence database (82896 sequences, 11/2014). Database searching was performed by using *S*-glutathionylation and S-carbamidomethylation as fixed Cys modifications, and Met oxidation and N-terminal-Gln->pyro-Glu conversion as variable modifications, a mass tolerance value of 2.0 Da for precursor ion and 0.8 Da for MS/MS fragments, trypsin as the proteolytic enzyme, and a missed cleavage maximum value of 2. Other MASCOT parameters were kept as default. Protein identification assigned with a minimum of 2 peptides with an individual ion score >30 and a *p*-value < 0.05, were further evaluated by comparison of the experimental mass and pI values obtained from 2-DE with their theoretical counterparts. Experiments were performed in technical duplicate on two biological replicates.

### 2.14. Immunohistochemical Analysis

Immunohistochemical (IHC) analyses were performed using a Ventana Autostainer Benchmark Ultra (Ventana Medical Systems, Inc., Tucson, AZ, USA), as previously described [9,20,23]. Briefly, histological sections (2 μm thick) of paraffin-embedded CCM surgical specimens, retrieved from the Department of Anatomy and Diagnostic Histopathology at the University Hospital “Città della Salute e della Scienza” of Turin, were deparaffinized, rehydrated, and incubated for 36 min in citrate buffer (pH 6.0) at boiling temperature for antigen retrieval. After blocking the activity of endogenous peroxidases by incubation with a H_2_O_2_ solution, histological sections were incubated at 37 °C for 32 min with 1:200 dilution of a mouse monoclonal antibody specific for GSH-protein complexes (ViroGen, Watertown, MA, USA). IHC labeling was developed by incubation for 5 to 10 min with 3,3′-diaminobenzidine (DAB) chromogen and substrate buffer containing H_2_O_2_, which results in a brown-colored precipitate at antigen site. Histological sections were then counterstained with hematoxylin, and digitized at 40× magnification by a Hamamatsu’s High-Resolution Nanozoomer S210 whole slide scanner (Hamamatsu Photonics, Arese, MI, Italy) [9,20,23].

The study was approved by the ethic institutional review board for “Biobanking and use of human tissue for experimental studies” of the Pathology Services (“Azienda Ospedaliera Città della Salute e della Scienza” of Turin, and Department of Medical Sciences of the University of Turin, Italy), and performed according to the standards of the Institutional Ethical Committee and the Helsinki Declaration. At the time of neurosurgery, an informed consent was asked by neurosurgeons to patients (or legal representatives) for scientific use of residual materials according to Institutional Rules defined by the Ethical Committee of the “Città della Salute e della Scienza” University Hospital, Turin. All patient records were anonymized and de-identified prior to analysis.

### 2.15. Statistical Analysis

Statistical analyses were performed using the Statistica Software (Ver 6.0, StatSoft, Tulsa, OK, USA). Analysis of variance (ANOVA) was applied for all parameters to test differences between different cell groups. Level of significance was set at *p* < 0.05, homogeneity of variance was analyzed by Cochram C, and the post hoc tests (Newman–Keuls) were used to discriminate between means of values. Multiple regression analyses were performed on antioxidant parameters to investigate possible correlations between various biological responses. 

### 2.16. Ethics Statement

Derivation and experimental use of KRIT1^−/−^ (K^−/−^) and KRIT1^+/+^ (K^9/6^) mouse embryonic fibroblast (MEF) cells followed the guidelines of the European Council Directive 86/609/EEC (24 November 1986) and Recommendation 2007/526/EC (18 June 2007), and were approved by the Ethics Committee on Laboratory Animal Care of the University of Torino (Torino, Italy), as previously reported [7].

## 3. Results

### 3.1. KRIT1 Loss-Of-Function Is Associated with Intracellular Glutathione Depletion

Since the original discovery that KRIT1 plays an important role in maintaining intracellular ROS homeostasis and limiting molecular and cellular oxidative dysfunctions [7], a growing body of evidence has shown that loss-of-function of this protein exerts pleiotropic effects on major redox-sensitive signaling pathways and mechanisms implicated in normal cell physiology and adaptive responses to oxidative stress and inflammation [3]. To further investigate the regulatory role of KRIT1 in cellular redox homeostasis and redox-sensitive mechanisms, we addressed the putative involvement of glutathione (GSH)—the most abundant cellular antioxidant and major modulator of the intracellular redox status. Indeed, reduced glutathione (GSH) is considered one of the most important ROS scavengers, and its ratio with oxidized glutathione (GSSG) contributes to the redox potential of the cell, and thereby to redox homeostasis. Conversely, a decrease in the GSH/GSSG ratio is generally associated with an increased cell susceptibility to oxidative stress, thereby serving as a marker of altered redox homeostasis and oxidative stress conditions underlying the etiology and progression of various human diseases, including vascular diseases [34,35,36,37].

**Figure 1:**
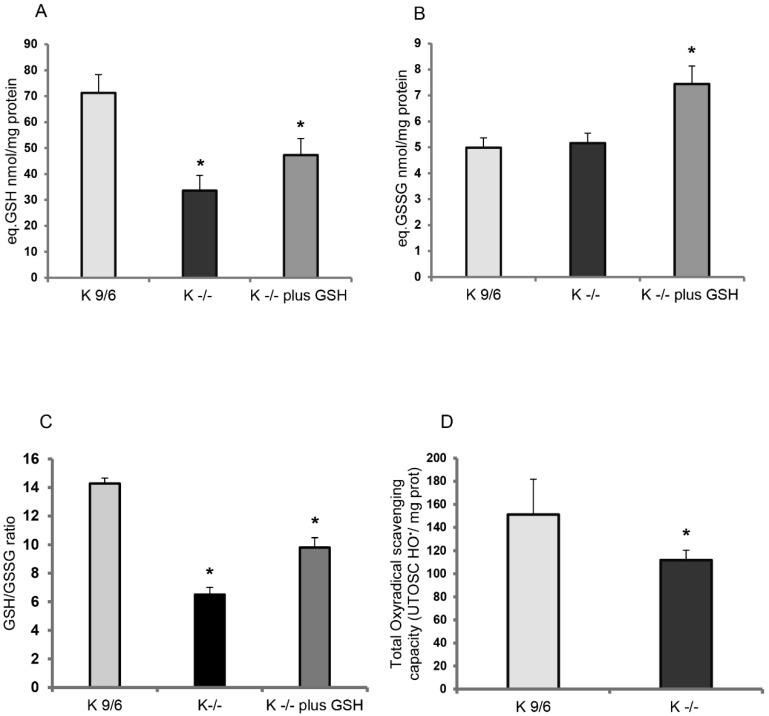
KRIT1 loss-of-function induces GSH depletion. (**A**,**B**) The amounts of total glutathione (GSH+GSSG in GSH equivalents) (A) and oxidized glutathione (GSSG) (B) were quantified in K^−/−^ cells left untreated or treated for 4 h with liposome-encapsulated GSH (5 mM final concentration) by an established enzymatic recycling assay, as described in Materials and Methods. K^9/6^ cells were used as control. (**C**) Histogram representing the GSH/GSSG ratio. (**D**) Total antioxidant capacity for peroxyl radicals in K^9/6^ and K^−/−^ cells, as determined by the total oxyradical scavenging capacity (TOSC) assay described in Materials and Methods. For all measurements, TOSC values were referred to protein concentration counterparts. Results are reported as mean values ± standard deviation (S.D.) of six different experiments. Asterisks above histogram bars indicate significant differences (*p* ≤ 0.05) between groups of means (post hoc comparison).

The GSH and GSSG levels and GSH/GSSG ratio were comparatively evaluated in K^−/−^ and K^9/6^ MEF cells, well-established cellular models that have previously allowed the identification of major KRIT1 functions and dysfunction effects [7,8,9,20,24,38]. In particular, total glutathione (GSH+GSSG) and oxidized glutathione (GSSG) were quantified by specific analytical methods, including an optimized recycling assay that spectrophotometrically measures the reduction of DTNB to TNB in the presence of glutathione reductase (GR) [39], as described in Materials and Methods. The outcomes of these analyses demonstrated that GSH levels were significantly lower in K^−/−^ as compared to K^9/6^ cells (Figure 1A,B), suggesting that KRIT1 loss-of-function is associated with a depletion of intracellular glutathione levels, which in turn is indicative of a compromised redox homeostasis. Consistently, the GSH/GSSG ratio resulted significantly diminished in K^−/−^ cells (Figure 1C), highlighting a detriment of the cellular antioxidant capacity and consequent shift of the intracellular redox state toward a more-oxidizing environment. Treatment of K^−/−^ cells with liposome-encapsulated GSH (5 mM final concentration for 4 h) determined a significant increase of GSSG levels along with the increase of total GSH (Figure 1A,B), indicating the immediate use of the GSH supplied, and confirming that depletion of cellular thiols, including GSH, contributes to the redox imbalance associated with KRIT1 loss-of-function. However, a significant rescue of the GSH/GSSG ratio was also observed (Figure 1C).

Given that cellular thiol content is the main determinant of total antioxidant capacity (TAC) [40], we then evaluated TAC levels in K^−/−^ and K^9/6^ cells using a total oxyradical scavenging capacity (TOSC) assay. As compared to K^9/6^ cells, TAC levels resulted significantly decreased in K^−/−^ cells (Figure 1D), suggesting that KRIT1 loss-of-function causes a concomitant depletion of cellular thiols and antioxidant defenses.

### 3.2. GSH Delivery Rescues the Cellular Redox Imbalance Induced by KRIT1 Loss-Of-Function

**Figure 2:**
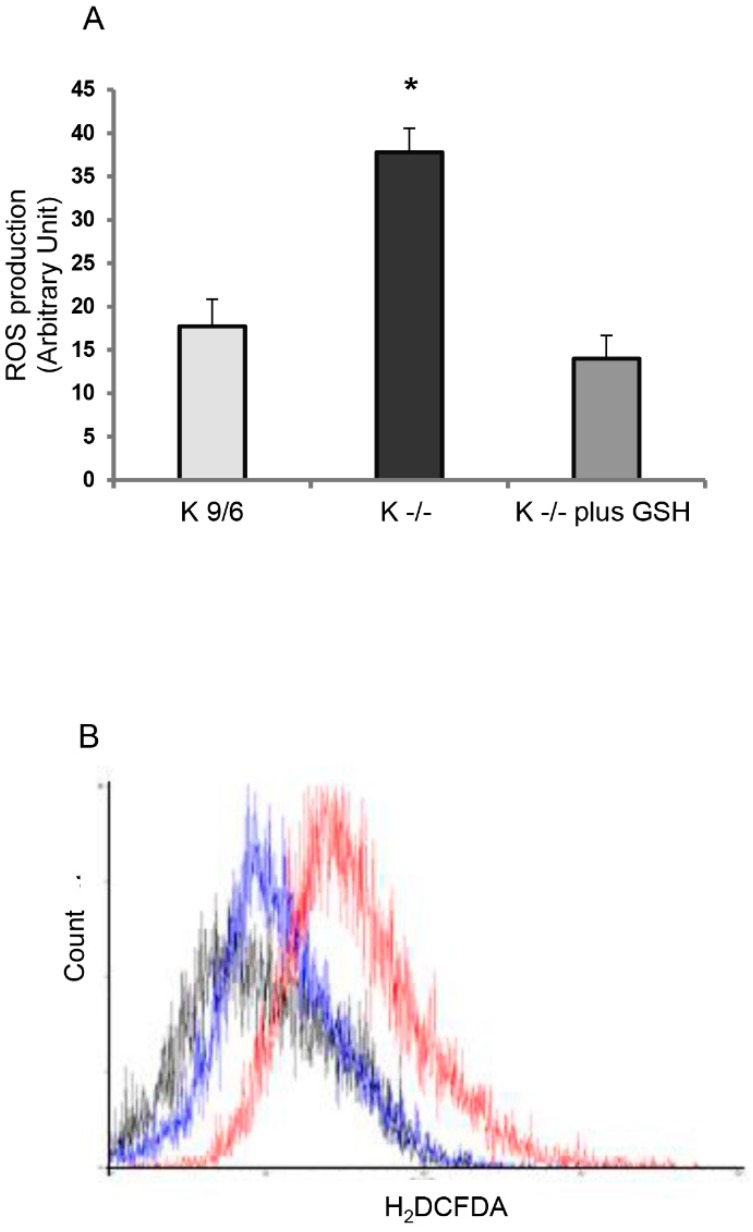
GSH delivery rescues the cellular redox imbalance induced by KRIT1 loss-of-function. (**A**) Measurement of ROS levels in K^−/−^ cells left untreated or treated for 4 h with 5 mM GSH delivered by liposomes. K^9/6^ cells were used as control. The cell-permeant carboxy-H_2_DCFDA (C400) probe was used for ROS labeling and measurements were performed by flow cytometry, as described in Materials and Methods. ROS concentrations are reported as fluorescence arbitrary units normalized to the initial mean control values, and represent the mean ± S.D. of three independent experiments. Asterisk indicates significant differences (*p* < 0.0001). (**B**) Flow cytometric graph representative of the data reported in (A). Gray line = K^9/6^ cells; red line = K^−/−^ cells; blue line = K^−/−^ cells plus GSH.

Previously, we demonstrated that KRIT1 loss-of-function causes a rise in intracellular ROS levels, thus affecting cellular redox homeostasis and redox-sensitive mechanisms [7,8]. To test whether this increase in ROS levels could be decreased by the administration of exogenous GSH, we treated K^−/−^ cells with 5 mM GSH delivered by liposomes, as described in Materials and Methods. The outcomes of these experiments demonstrated that the enhanced ROS levels observed in K^−/−^ cells decreased significantly after GSH administration (Figure 2A,B), suggesting that exogenous GSH can attenuate the redox imbalance associated with KRIT1 loss-of-function.

### 3.3. KRIT1 Loss-Of-Function Affects the Activity of GSH-Dependent Antioxidant Enzymes

Under basal conditions, the adverse effects of ROS are prevented by a wide array of low molecular weight scavengers (including GSH) and antioxidant enzymes, which interact in a sophisticated network to determine an equilibrium condition. In order to assess whether GSH-dependent antioxidant enzymes were affected by KRIT1 loss-of-function, we comparatively evaluated their basal activity in K^−/−^ and K^9/6^ cells. In particular, we analyzed the activity of glutathione S-transferases (GSTs), glutathione peroxidases (GPXs), and glutathione reductase (GR), as well as of catalase (CAT).

**Figure 3:**
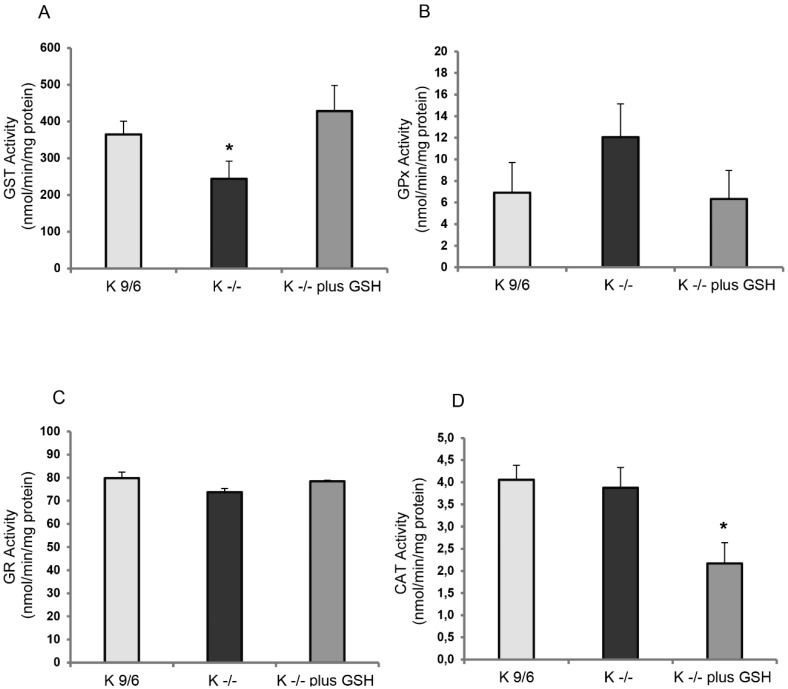
KRIT1 loss-of-function affects the activity of GSH-dependent antioxidant enzymes. Glutathione-S-transferase (GST) (**A**), glutathione peroxidase (GPX) (**B**), glutathione reductase (GR) (**C**), and catalase (CAT) activity (**D**) in K^9/6^ and K^−/−^ cells left untreated or treated for 4 h with 5 mM GSH encapsulated within liposomes was measured by specific enzymatic assays, as described in Materials and Methods. Results are reported as mean values ± S.D. of six different experiments. Asterisks above histogram bars indicate significant differences (*p* ≤ 0.05) between groups of means (post hoc comparison).

Glutathione S-transferases (EC 2.5.1.18) constitute a superfamily of soluble isoenzymes performing detoxification, transporting, and antioxidant functions. These proteins catalyze the conjugation of an electrophilic substrate to reduced GSH to facilitate the cellular elimination of exogenous substances. Total GST activity was examined in K^−/−^ and K^9/6^ cells using a colorimetric assay with a broad range of isozyme detectability (e.g., alpha, mu, pi, and other GST isoforms), as described in Materials and Methods. The outcomes showed a significant decrease of GST activity in K^−/−^ cells as compared to K^9/6^ cells (Figure 3A), which was largely rescued by pretreatment of K^−/−^ cells with liposome-encapsulated GSH (Figure 3A, K^−/−^ plus GSH), suggesting that the depletion of GST activity in K^−/−^ cells may be related to GSH availability. Accordingly, some GSTs reduce lipid hydroperoxides and xenobiotics by oxidizing GSH to GSSG.

Glutathione peroxidases (EC 1.11.1.9) are a family of isoenzymes involved in detoxification of lipid hydroperoxide and reactive nitrogen species through a cyclical reaction that converts reduced glutathione (GSH) to oxidized glutathione (GSSG) [41]. GPX activity was evaluated in K^−/−^ and K^9/6^ cells using an assay that detects both Se-dependent and Se-independent isoenzymes. As compared to K^9/6^ cells, an increased GPX activity was observed in K^−/−^ cells (Figure 3B), which was largely rescued by pretreatment of K^−/−^ cells with liposome-encapsulated GSH (Figure 3B, K^−/−^ plus GSH). Although not reaching statistical significance, these results indicate a tendency towards a potential correlation between KRIT1 loss-of-function and increased GPX activity.

Glutathione reductase (GR) (EC 1.6.4.2) utilizes NADPH to convert oxidized glutathione (GSSG) to reduced glutathione (GSH) [42]. Despite not being a primary antioxidant enzyme, GR is essential to maintain the correct GSH/GSSG ratio and, consequently, the intracellular redox status. GR activity was evaluated in K^−/−^ and K^9/6^ cells using an assay that evaluates the consumption of NADPH during the reduction of GSSG. The outcomes demonstrated that GR activity was comparable in K^−/−^ and K^9/6^ cells, and did not change upon cell treatment with liposome-encapsulated GSH (Figure 3C), suggesting that KRIT1 loss-of-function does not affect GR activity. 

Catalase (CAT) (EC 1.11.1.6) neutralizes H_2_O_2_ into H_2_O and O_2_, thus detoxifying this ROS and preventing the occurrence of the Fenton reaction, which converts H_2_O_2_ into highly reactive hydroxyl radicals. CAT activity was evaluated in K^−/−^ and K^9/6^ cells using a specific assay. Consistent with previous findings [7], no significant differences were observed in CAT activity between K^9/6^ and K^−/−^ cells. In contrast, a significant reduction of CAT activity was observed in K^−/−^ cells upon treatment with liposome-encapsulated GSH (Figure 3D), suggesting a potential adaptive response.

Overall, these results point to a major impact of KRIT1 loss-of-function on the activity of GSH-dependent antioxidant enzymes, including GSTs and GPXs, which is most likely attributable to its effect on intracellular GSH levels as it can be reversed by cell treatment with exogenous GSH.

### 3.4. KRIT1 Loss-Of-Function Induces Changes in Protein S-Glutathionylation Pattern

*S*-glutathionylation—the reversible formation of mixed disulfide bonds between GSH or GSSG and redox-sensitive cysteine residues within proteins—has emerged as a central mechanism by which changes in the intracellular redox status may be transduced into functional responses [13,16,43,44]. In particular, accumulating evidence demonstrates that *S*-glutathionylation of reactive cysteines occurs as a consequence of the decreased GSH/GSSG ratio and the increased formation of transient cysteine oxidation products, such as sulfenic acid intermediates, caused by oxidative stress, thus playing a central role in protecting fundamental redox-sensitive proteins against irreversible oxidation, and influencing a number of critical redox signaling events [45]. Consistently, reversible protein-*S*-glutathionylation (PSSG) is emerging as a major thiol-based redox switch for dynamic, post-translational regulation of the stability and activity of a variety of structural and regulatory target proteins involved in adaptive cellular responses to oxidative stress [13,16,43,44,46,47].

**Figure 4:**
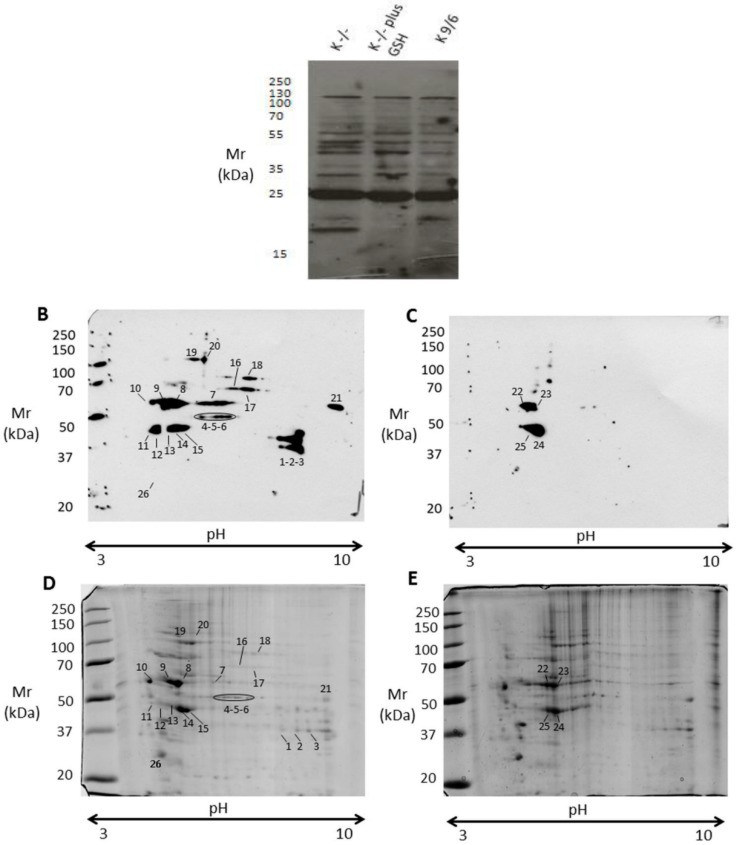
KRIT1 loss-of-function induces changes in protein *S*-glutathionylation pattern. Total protein extracts of K^−/−^ cells left untreated or treated for 4 h with 5 mM GSH encapsulated within liposomes were separated by monodimensional (**A**) and two-dimensional electrophoresis (**B**–**E**) under nonreducing conditions, and analyzed by immunoblotting with an anti-GSH antibody to detect protein *S*-glutathionylation adducts (A–C), or by colloidal Coomassie staining to identify protein spots by mass spectrometry analysis (D,E). K^9/6^ cells were used as control. (A) Equal amounts of protein extracts (30 μg) of the indicated cells were analyzed by SDS-PAGE and Western blotting under nonreducing conditions. (B–E) Equal amounts of protein extracts (200 μg) of K^−/−^ (B,D) and K^9/6^ (C,E) cells were analyzed by two-dimensional (2-D) electrophoresis and Western blotting. Parallel experiments were performed for Western blotting (B,C) and colloidal Coomassie staining (D,E) analyses. Digitalized images of PVDF membranes and colloidal Coomassie-stained gels were acquired and analyzed with a dedicated software that ensured spot matching and relative quantitation. Gel spots corresponding to immunoreactive signals were manually excised from 2D-gels and further subjected to mass spectrometric analysis. Experiments were performed in technical duplicate on two biological replicates.

The increased levels of GSSG and decreased ratio of GSH to GSSG observed in K^−/−^ cells clearly indicated a cellular redox imbalance toward a mild oxidative stress condition, raising the possibility that changes in PSSG pattern may also occur. To verify this possibility, the formation of protein-GSH mixed disulfides was examined in K^−/−^ and K^9/6^ cells by a comparative immunoblotting analysis of total cell extracts with an anti-GSH antibody. Along with *S*-glutathionylated proteins present in K^9/6^ cell extracts, immunoblotting analysis of K^−/−^ cell extracts showed some novel *S*-glutathionylated protein bands (Figure 4A), which were abolished by cell pretreatment with a GSH supply (Figure 4A, K^−/−^ plus GSH), suggesting that the changes in PSSG associated with KRIT1 loss-of-function are redox-dependent and reversible upon GSH supplementation.

To further investigate and identify proteins undergoing differential *S*-glutathionylation in K^−/−^ versus K^9/6^ cells, protein extracts were separated by two-dimensional polyacrylamide gel electrophoresis (2-D PAGE) and analyzed by mass spectrometry, as described in Materials and Methods. Analysis of 2-D blots with an anti-GSH antibody showed a higher number of *S*-glutathionylated proteins in K^−/−^ cells (Figure 4B) compared to K^9/6^ cells (Figure 4C), suggesting that KRIT1 loss-of-function causes a significant increase in *S*-glutathionylation of several proteins. To identify these *S*-glutathionylated proteins, digitized images from Western blotting were matched to counterparts from colloidal Coomassie-stained gels (Figure 4D,E), and protein spots corresponding to immunoreactive signals were excised and subjected to mass spectrometry analysis. Twenty out of twenty-six analyzed protein spots were unequivocally identified, as reported in Table 1.

**Figure 5:**
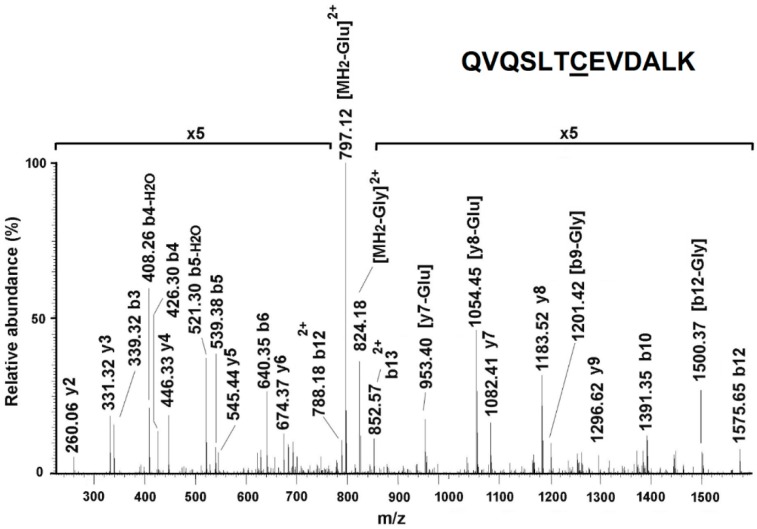
Identification of *S*-glutathionylated cysteine 328 in vimentin. Collision induced dissociation fragmentation spectrum of the doubly charged (*m*/*z* 862.3) modified peptide (322–334) from vimentin-bearing *S*-glutathionylated Cys328. This peptide was isolated in spot 10 from K^−/−^ cells (Figure 4B,E). Assigned fragments are reported in the spectrum, together with the peptide sequence where the modified residue is underlined. Glu, glutathione.

The outcomes of these analyses demonstrated that KRIT1 loss-of-function has a striking impact on the pattern of protein *S*-glutathionylation. Indeed, most of the immunoreactive signals in K^−/−^ cells had no counterparts in K^9/6^ cells, and corresponded mainly to proteins involved in key processes of cellular homeostasis, stress response, and adaptation, including redox-sensitive enzymes of the energy metabolism, chaperonins, and cytoskeletal proteins. In particular, proteins that appeared *S*-glutathionylated only in K^−/−^ cells were glyceraldehyde-3-phosphate dehydrogenase (GAPDH) (spots 1, 2 and 3), alpha-enolase (ENO1) (spots 4, 5, and 6), 60 kDa heat shock protein (HSP60) (spot 7), calreticulin (CALR) (spot 10), creatine kinase B-type (CKB) (spots 11 and 12), tubulin beta-4B chain (TUBB4B) (spot 13), and tropomyosin isoforms 3/4 (TPM3/4) (spot 26) (Figure 4D,E and Table 1). A careful comparison of Western blotting images from K^−/−^ and K^9/6^ cells also evidenced a significant change in the signal intensity of several spots. In particular, a protein whose *S*-glutathionylation appeared increased in K^−/−^ cells was vimentin (Figure 4; spots 8, 9, 22, and 23), whose modified cysteine was identified by tandem mass spectrometry (Figure 5). Moreover, a significant increase in *S*-glutathionylation was also observed for actin (ACT) (spots 14, 15, 24, and 25) (Figure 4D,E and Table 1).

In order to verify if any evidence of *S*-glutathionylation had been already reported for the proteins identified in this study, we searched the dbGSH repository, which integrates experimentally verified *S*-glutathionylation sites from multiple species. Consistent with our experimental results, we found evidence that *S*-glutathionylation exists in mouse for all the proteins reported in Table 1, including a clear coincidence for the modified Cys328 identified in vimentin, with the unique exception of tubulin beta-4B chain, which nevertheless was found as *S*-glutathionylated in humans. In addition, further support to our experimental results was provided by previous reports showing that all the *S*-glutathionylated proteins identified in our study can indeed undergo *S*-glutathionylation in several cells under varying conditions of oxidative stress [45,46].

Overall, our findings show that loss-of-function of KRIT1 leads to *S*-glutathionylation of distinct structural, metabolic, and regulatory proteins involved in stress responses and adaptation, suggesting important physiopathological implications. Further studies focused on detailed molecular validation and functional characterization of the *S*-glutathionylation of these proteins should provide additional insights into its putative role in molecular mechanisms of CCM disease pathogenesis.

### 3.5. Confirmation of HSP60 S-Glutathionylation Induced by KRIT1 Loss-Of-Function

**Figure 6:**
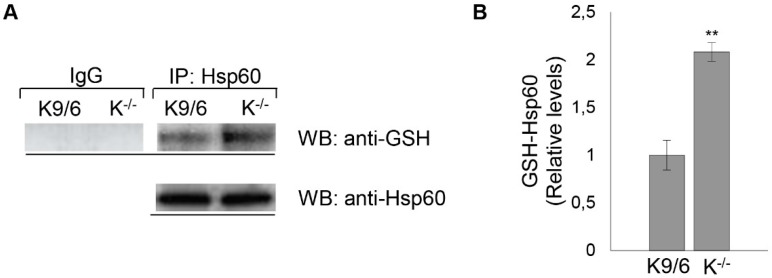
Confirmation of HSP60 *S*-glutathionylation induced by KRIT1 loss-of-function. (**A**) Lysates from K^9/6^ and K^−/−^ cells were immunoprecipitated with protein G agarose-coupled anti-HSP60 (IP: Hsp60) and subjected to Western blotting (WB) with anti-GSH antibody. Blots were then stripped and reprobed with anti-HSP60 antibody to ensure equal immunoprecipitation of HSP60 proteins. Mouse IgG was used as negative control for immunoprecipitation. (**B**) The histogram indicates mean (±S.E., *n* = 3) levels of GSH-Hsp60 relative to total Hsp60 in IP samples, as quantified by densitometric analysis of WB bands. Normalized optical density values were expressed as relative protein level units. Asterisks indicate significant differences versus K^9/6^ cells (*p* ≤ 0.01).

Among the differentially *S*-glutathionylated proteins identified by nLC-ESI-LIT-MS/MS analysis in K^−/−^ cells was the heat shock protein HSP60: a mitochondrial chaperonin that plays a major role in the correct folding and macromolecular assembly of many proteins imported into the mitochondria and is essential in maintaining mitochondrial integrity and resistance to oxidative stress [48]. Remarkably, this important mitochondrial protein was recently implicated in Nrf2-mediated adaptive responses and enhanced cell sensitivity to oxidative stress consequent to KRIT1 loss-of-function [20], suggesting that its *S*-glutathionylation may be related to these effects. To confirm with a complementary approach that indeed KRIT1 loss-of-function causes *S*-glutathionylation of HSP60, lysates of K^−/−^ cells were analyzed by immunoprecipitation with an HSP60 antibody followed by Western blot with an anti-GSH antibody. The results of these analyses demonstrated that the HSP60 protein was *S*-glutathionylated at high levels in K^−/−^ cells, thus validating the nLC-ESI-LIT-MS/MS analysis outcome (Figure 6A,B). However, some *S*-glutathionylation of HSP60 was also observed in K^9/6^ cells, although at much lower levels (Figure 6A,B), suggesting that the immunoprecipitation and Western blotting (IP/WB) method used as a confirmatory test was more sensitive quantitatively than the 2-D PAGE immunoassays for detection of *S*-glutathionylated proteins in total cell extracts reported above (Figure 4B,C).

Taken together, the combined 2D-PAGE/mass spectrometry and IP/WB approaches demonstrated unequivocally that KRIT1 loss results in a significant increase in the basal level of *S*-glutathionylation of HSP60, suggesting potential implications in abnormal adaptive responses to KRIT1 loss-of-function reported previously [20]. While future focused studies are required to address the role of increased *S*-glutathionylation in HSP60 function, our finding provides further insights into the effects of altered redox homeostasis caused by KRIT1 dysfunctions, and opens a novel research avenue for a deep characterization of the role of HSP60 in molecular mechanisms underlying CCM disease pathogenesis. 

### 3.6. Redox-Dependent Changes in PSSG Occur in Human Brain Microvascular Endothelial Cells upon KRIT1 Knockdown

**Figure 7:**
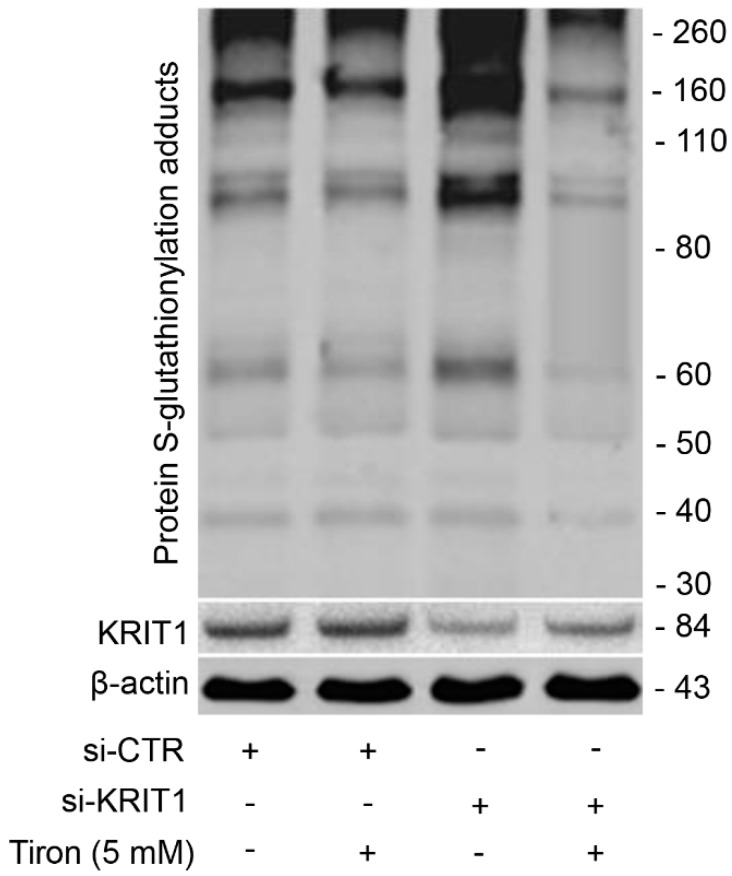
Redox-dependent changes in PSSG occur in human brain microvascular endothelial cells upon KRIT1 knockdown. Human brain microvascular endothelial cells (hBMEC) grown under standard conditions were transfected with either KRIT1-targeting siRNA (si-KRIT1) or a scrambled control (si-CTR). Cells were then either left untreated or treated with Tiron, lysed, and analyzed by Western blotting under nonreducing conditions with an anti-GSH antibody to detect protein *S*-glutathionylation adducts, and then compared with KRIT1 protein expression levels. β-actin was used as internal loading control for Western blot normalization. Results are representative of three separate experiments. Notice that KRIT1 knockdown in human brain microvascular endothelial cells leads to a significant increase in the levels of protein *S*-glutathionylation adducts, which are significantly reverted by cell treatment with the ROS scavenger Tiron.

Growing evidence indicates that redox-dependent changes in PSSG play important roles in endothelial biology. Indeed, *S*-glutathionylation of distinct proteins in endothelial cells is emerging as a novel redox mechanism of vascular barrier dysfunction implicated in the etiology of human diseases [49,50]. To investigate whether the upregulation of PSSG adducts observed in K^−/−^ MEF cells could be recapitulated in a different cellular model, more strictly connected to CCM disease, we performed KRIT1 knockdown in human brain microvascular endothelial cells (hBMEC) according to a previously optimized siRNA-based procedure [9,20]. In line with experimental observations in K^−/−^ MEF cells, we found that PSSG adducts were significantly upregulated in KRIT1-silenced versus control hBMEC cells (Figure 7), showing that the upregulation of PSSG adducts induced by the loss-of-function of KRIT1 can occur in brain microvascular endothelial cells. Furthermore, and remarkably, this effect was significantly rescued by cell treatment with the antioxidant Tiron, a mitochondria-permeable ROS scavenger previously shown to be effective in rescuing major molecular hallmarks of KRIT1 dysfunctions and CCM disease [20,23], demonstrating that the accumulation of PSSG adducts is indeed a reversible, redox-dependent phenomenon triggered by KRIT1 loss-of-function (Figure 7).

### 3.7. Enhanced Protein S-Glutathionylation Occurs in Endothelial Cells Lining Human CCM Lesions

Previously, we demonstrated that markers of altered redox homeostasis and signaling, including proteins involved in either pro-oxidant or antioxidant mechanisms, are upregulated in the endothelium of human CCM vessels [8,9,20,23]. Thereby, to examine the clinical relevance of our novel findings demonstrating the upregulation of PSSG adducts in cellular models of CCM disease, we analyzed PSSG levels in human CCM lesions. Indeed, whereas protein *S*-glutathionylation is emerging as a critical redox signaling mechanism in cardiovascular physiopathology [49,50,51], significant increases in PSSG levels have been found in human diseases associated with oxidative stress, and indicated as possible biomarkers of the evolution of the disease, with the consequent attribution of diagnostic/prognostic importance [17,44,46].

**Figure 8:**
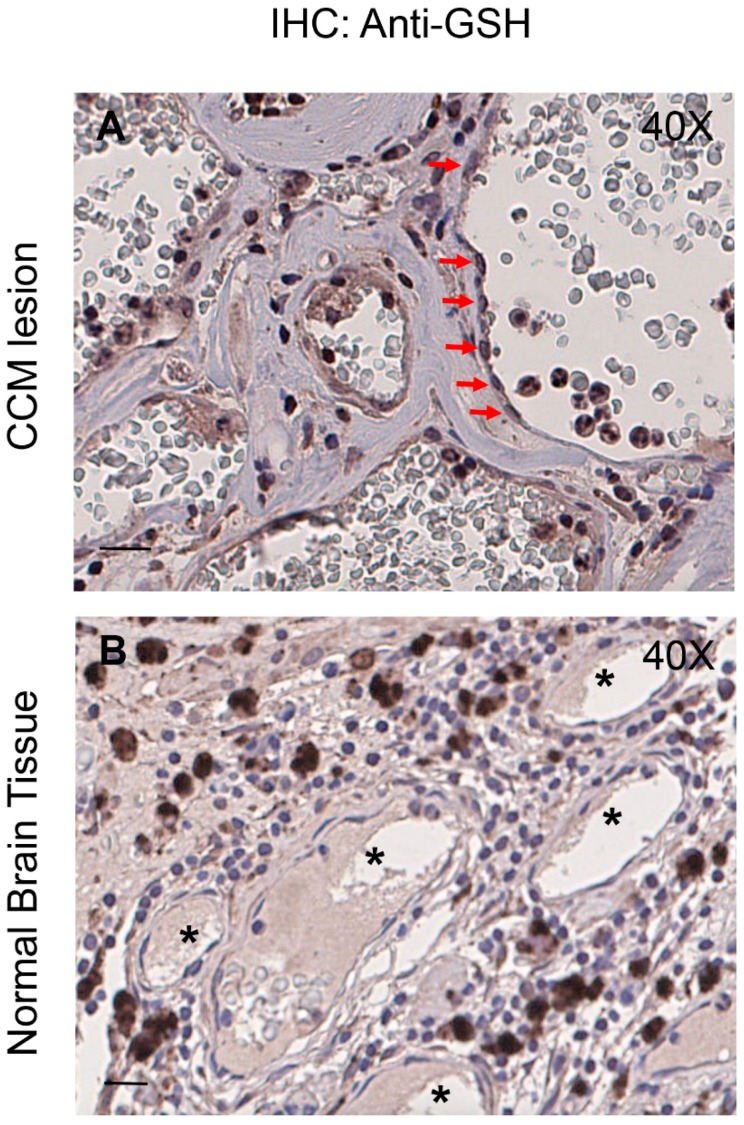
Increased levels of *S*-glutathionylated proteins occur in endothelial cells lining human CCM lesions. Immunohistochemical (IHC) analysis of *S*-glutathionylated proteins in histological sections of a representative human CCM surgical specimen containing a cluster of CCM vessels (**A**) and perilesional normal vessels serving as internal negative controls (**B**). Scale bar: 200 µm. Notice that a significant positive IHC staining for *S*-glutathionylated proteins is evident in endothelial cells lining the lumen of CCM lesions (A, arrows), as compared with endothelial cells lining the lumen of perilesional normal vessels (B, asterisks).

Histological samples of human CCM lesions were obtained from archived paraffin-embedded surgically resected CCM specimens [9,20], and PSSG levels were evaluated by immunohistochemical (ICH) studies with a monoclonal antibody that recognizes GSH-protein complexes (see Materials and Methods). IHC analysis of three distinct CCM specimens from KRIT1 loss-of-function mutation carriers with confirmed diagnosis of CCM revealed increased levels of PSSG adducts in endothelial cells lining the lumen of abnormally dilated CCM vessels (Figure 8A,B) as compared with perilesional normal vessels (Figure 8C,D), demonstrating that the enhanced protein *S*-glutathionylation caused by KRIT1 loss-of-function occurs also in vivo, suggesting a potential relationship with CCM disease. Remarkably, the staining with the anti-GSH antibody was significant mainly in endothelial cells lining large CCM caverns (Figure 8A), suggesting a potential correlation with the phenotypic distribution of CCM lesion severity.

Taken together with the findings in cellular models, the outcomes of IHC analyses on surgical samples of human CCM lesions suggest that the observed enhanced protein *S*-glutathionylation could be part of an abnormal adaptive response to altered redox homeostasis and signaling caused by KRIT1 loss-of-function, thus representing a novel pathological signature in CCM disease that might be developed for diagnostic and therapeutic strategies in the future.

## 4. Discussion

Great progress has been made during the last decade in understanding the biological roles of the three known CCM proteins—KRIT1, CCM2, and CCM3—and their role in the pathogenesis of cerebral cavernous malformation (CCM) disease, revealing stunning complexity [11]. In fact, loss-of-function of CCM proteins has been demonstrated to affect multiple cellular structures and mechanisms involved in fundamental physiopathological processes [52], including cadherin-mediated cell–cell junctions [53,54], integrin-mediated cell-matrix adhesion [55,56,57], and RhoA-mediated actin cytoskeleton organization and dynamics [58,59,60,61]. Consistent with their emerging pleiotropic roles, CCM proteins have been shown to modulate the functions of a plethora of signaling molecules and pathways that influence almost all aspects of endothelial biology, including vascular development, maintenance of endothelial cell homeostasis and barrier function, and responses to a variety of physiological and pathological stimuli (reviewed in Retta and Glading [3]). In particular, KRIT1, whose loss-of-function mutations are responsible for over 50% of the familial cases of CCM disease, has been shown to ensure homeostasis of endothelial cells and inhibit abnormal angiogenic responses by mediating Rap1-induced stabilization of endothelial cell–cell junctions [53,62]; activating the Delta-Notch signaling axis [63,64]; triggering the integrin-linked kinase survival pathway [65]; inhibiting RhoA/ROCK activation [58,59], vascular endothelial growth factor (VEGF) signaling [38], beta1 integrin-dependent endothelial contractility, and fibronectin remodeling [55]; preventing β-catenin and transforming growth factor beta (TGFβ) signaling-driven endothelial-to-mesenchymal transition (EndMT) [66,67]; and limiting activation of signaling pathways involving the mechanosensitive transcription factors Kruppel-like factor 2 and 4 (KLF2/4) [68,69,70,71].

Despite these great advances, the mechanistic interconnection of the multiple molecules and mechanisms that have been linked to KRIT1 so far, as well as their causal role in CCM pathogenesis, have remained elusive. Indeed, studies in animal models have clearly indicated that homozygous loss of KRIT1 is not fully sufficient to cause CCM lesion formation and disease progression, suggesting the necessary contribution of additional triggers, including microenvironmental stress factors [3]. Accordingly, accumulating evidence in cellular and animal models demonstrates that the pleiotropic functions of KRIT1 can be largely attributed to its emerging role in the modulation of cellular redox homeostasis and signaling, suggesting a potential unifying molecular mechanism, and raising the possibility that altered redox homeostasis and increased susceptibility to oxidative stress are primarily implicated in the pathogenesis of CCM disease [3,11]. Specifically, KRIT1 has been clearly involved in redox-sensitive mechanisms and signaling pathways that maintain normal cellular homeostasis both under physiological conditions and in response to oxidative stress and inflammation, including autophagy and signaling pathways regulated by redox-sensitive kinases and transcription factors [6,7,8,9,20,24,72]. Consistent with these findings, the wide variety of mechanisms attributed to CCM formation, including downregulation of cell–cell contacts, altered cell-matrix adhesion, activation of RhoA signaling and cytoskeleton contractility, and endothelial-to-mesenchymal transition, all fit comfortably under the umbrella of known redox-sensitive mechanisms [3].

To shed more light on this intricate mechanistic scenario, here we addressed the possibility that KRIT1 loss-of-function affects additional redox-sensitive mechanisms, including the glutathione (GSH) redox system. Indeed, this system is known to play a major role in cellular redox homeostasis and antioxidant defenses, and has been clearly implicated in cerebral microvascular endothelial biology and pathobiology, exerting a protective role against oxidative disruption of endothelial barrier function and oxidative stress-associated neurovascular disorders [35,36]. Moreover, it can influence inflammatory and angiogenic responses [73,74]. Furthermore, and importantly, it is also implicated in the modulation of protein activity and function by *S*-glutathionylation, a reversible oxidative post-translational modification (OPTM) involved in redox regulation and signaling, which is now actively investigated as possible biomarker of human diseases associated with oxidative stress, including cardiovascular diseases [15,17,18,46,48,51,75].

The intracellular concentration of GSH, which usually range from 1 to 10 mM, and the ratio of GSH to its oxidized form, glutathione disulfide (GSSG), reflect the redox potential of the cell and thereby influence redox homeostasis, whereas their decrease is generally considered a marker of oxidative stress. Accordingly, under normal conditions, the GSH/GSSG ratio is closely regulated and maintained at high levels, exceeding 100:1, while in various models of oxidative stress, this ratio has been demonstrated to decrease to values of 10:1 and even 1:1 [28,37]. Furthermore, it is now established that decreased GSH levels and GSH/GSSG ratio correlate with increased cellular susceptibility to oxidative stress and can contribute to some human diseases, including cardiovascular diseases [28,34].

Taking advantage of KRIT1^−/−^ MEF cells, an established cellular model that allowed the identification of new molecules and mechanisms involved in CCM disease [7,8,9,38,72,76,77,78], we found that KRIT1 loss causes a significant decrease in total GSH and increase in GSSG levels, with a consequent deficit in the GSH/GSSG redox ratio and GSH-mediated antioxidant capacity.

Moreover, we observed also a significant reduction in glutathione-S-transferase (GST) activity, which catalyzes the conjugation of GSH to a wide variety of endogenous and exogenous electrophilic compounds to aid in their detoxification, thereby contributing in protecting cells from oxidative damage [79]. Notably, all these molecular alterations could be rescued by providing cells with exogenous GSH, suggesting that they were interconnected with alterations in the thiol-redox homeostasis. Indeed, they were consistent with our previous findings demonstrating that KRIT1 loss-of-function causes intracellular redox imbalance and sustained mild oxidative stress conditions [7,8,9,20], suggesting that changes in the GSH redox status and GST activity contribute to these effects.

Furthermore, using a proteomic approach we found a significant increase in the level of global protein *S*-glutathionylation (PSSG), which is in turn consistent with a decreased GSH/GSSG ratio and increased intracellular oxidation state. Accordingly, PSSG is an important mechanism for dynamic oxidative stress-induced post-translational modification of a variety of regulatory, structural, and metabolic proteins, which takes place by a reversible thiol–disulfide exchange between protein -SH groups and GSSG, and plays a major role in protecting target proteins against irreversible oxidation of critical cysteine residues, as well as in influencing their structural and functional properties. Like protein phosphorylation, PSSG can in fact modulate enzyme activities, alter transcription profiles, modify protein–protein interactions, and regulate adaptive cell signaling [13]. Indeed, PSSG is attracting growing interest as both a biomarker of oxidative stress and a biological switch involved in a number of critical oxidative signaling events through the modulation of several structural and regulatory proteins, including cytoskeletal proteins, enzymes (e.g., kinases and phosphatases), chaperonins, and transcription factors [13,44].

Importantly, a significant increase in the global level of PSSG adducts was also observed in KRIT1-silenced human brain microvascular endothelial cells (hBMEC), and could be rescued by cell treatment with the antioxidant Tiron, showing that KRIT1 loss-of-function results in a redox-dependent and reversible enhancement of protein *S*-glutathionylation in different cell types. In addition, elevated levels of PSSG adducts were also detected in endothelial cells lining human CCM lesions, suggesting that they may represent a novel pathological signature and possible biomarkers of oxidative stress correlated with CCM disease onset and progression.

Redox proteomic analysis by nLC-ESI-LIT-MS/MS allowed the identification of twenty proteins that were differentially *S*-glutathionylated in KRIT1 null vs. KRIT1 expressing cells. Among these, there were members of the chaperonin family, such as heat shock protein 60 (HSP60) and calreticulin (CALR), the glycolytic enzymes glyceraldehyde-3-phosphate dehydrogenase (GAPDH) and alpha-enolase (ENO1), creatine kinase B-type (CKB), and important cytoskeletal proteins, including actin, tubulin beta-4B chain, tropomyosin, and vimentin. Remarkably, all these proteins are known to be redox-sensitive and involved in biological processes associated with cellular redox homeostasis and the adaptive response to oxidative stress, including the unfolded protein response (UPR), redox signaling, energy metabolism, and cytoskeleton organization and dynamics. Moreover, all were previously shown to be subjected to ROS-induced *S*-glutathionylation in various cell types, including endothelial cells [16,44], and in distinct subcellular compartments related to redox functions, including mitochondria and endoplasmic reticulum [80,81], suggesting that their *S*-glutathionylation is part of the overall cellular adaptive response to altered redox homeostasis associated with KRIT1 loss-of-function. Accordingly, enhanced *S*-glutathionylation of chaperonins, such as HSP60 and CALR, glycolytic enzymes, including GAPDH and ENO1, and controllers of cellular energy homeostasis, such as CKB and cytoskeletal proteins, represents a common signature of mild oxidative stress conditions [16,44]. 

While all the differentially *S*-glutathionylated proteins identified by 2D-PAGE/mass spectrometry analyses deserve further validation by complementary approaches, such as IP/WB analysis, we directed our initial attention to the heat shock protein HSP60: a mitochondrial protein essential for preventing denaturation and aggregation of many mitochondrial proteins under various stress conditions, including both endogenous and exogenous oxidative stress [48]. In fact, besides being directly involved in conferring resistance against oxidative stress, members of the HSP chaperonin family have been recently implicated in abnormal adaptive responses and enhanced cell sensitivity to oxidative stress consequent to KRIT1 loss-of-function [20]. Combining 2D-PAGE/mass spectrometry and IP/WB approaches, we confirmed that KRIT1 loss-of-function indeed causes a significantly increased *S*-glutathionylation of HSP60, raising the possibility that this effect has important physiopathological implications.

Further focused studies are required to assess whether PSSG events associated with KRIT1 loss-of-function, including *S*-glutathionylation of HSP60, only represent a primary defense mechanism against irreversible oxidative damage of fundamental regulatory and structural proteins, or they also exert a role in redox modulation of protein activity/function and signal transduction. Nevertheless, accumulated evidence suggests that *S*-glutathionylation of the redox-sensitive chaperonins, glycolytic enzymes, and cytoskeletal proteins identified in our study may serve as both protective and regulatory mechanism by which cells respond coordinately, effectively, and reversibly to redox changes and oxidative insults [16,44,82]. In particular, while the role of basal *S*-glutathionylation in HSP60 function remains unclear, it has been suggested that increased *S*-glutathionylation of HSP60 may either potentiate its protective chaperoning activity [16,83] or enhance cell susceptibility to oxidative stress [84], likely depending on cell type and context. Indeed, HSP60 has been shown to reside also in extramitochondrial compartments, including extracellular space, cytosol, and nucleus, where it may exert a wide range of functions dependent or independent of its chaperoning activity [85,86]. Moreover, *S*-glutathionylation of CALR, a highly conserved endoplasmic reticulum calcium-binding chaperone protein, has been shown to influences cell sensitivity to drug-induced unfolded protein response [87]. Furthermore, it has been reported that *S*-glutathionylation of GAPDH, ENO1, and CKB is likely inhibitory, and might represent an adaptive and protective homeostatic response to an excess of oxidative challenges, including those occurring in pathological conditions associated with oxidative stress, such as cardiovascular pathologies [16,82,88,89,90]. Finally, our finding that redox changes associated with KRIT1 loss-of-function result in enhanced *S*-glutathionylation of major cytoskeletal proteins is consistent with clear evidence that PSSG is implicated in the redox regulation of a variety of cytoskeletal proteins, including actin, tubulin, vimentin, myosin, and tropomyosin, regulating actin microfilament and microtubule dynamics in both physiological and pathological contexts [44,82]. Indeed, increasing evidence indicates that reversible *S*-glutathionylation of specific cysteine residues in cytoskeletal proteins is relevant to their function by either protecting them against irreversible oxidation or modulating their supramolecular organization, thus contributing to the cell’s general adaptive response to altered redox homeostasis and oxidative stress [82]. On the other hand, it is now clear that redox regulation of the cytoskeleton plays a critical role in the vascular system, serving as a pivotal modulator of endothelial cell homeostasis and barrier function, with a consequent significant impact on vascular diseases [91], suggesting a potential implication in CCM disease.

## 5. Conclusions

**Figure 9:**
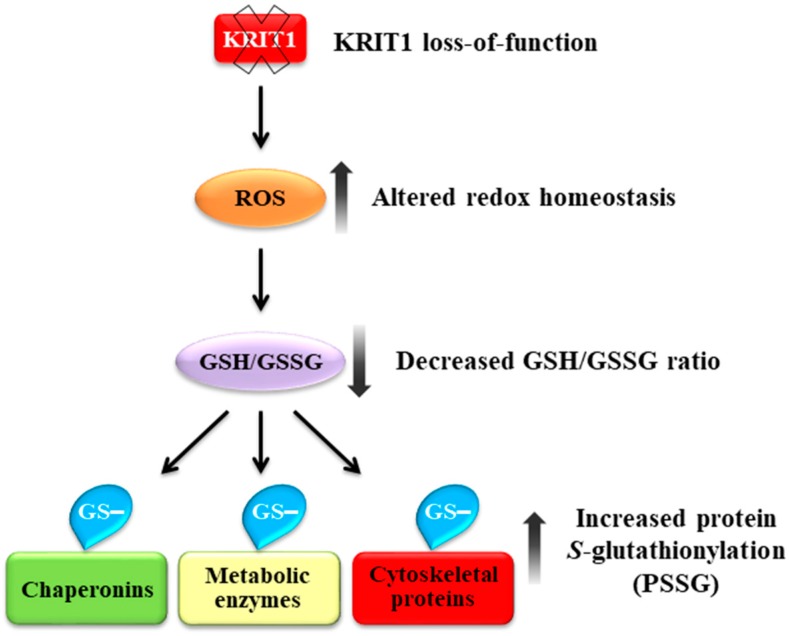
Schematic model representing the *S*-glutathionylation of distinct structural and regulatory proteins as a novel molecular signature of KRIT1 loss-of-function. The altered intracellular redox homeostasis caused by KRIT1 loss-of-function affects the glutathione (GSH) redox system, leading to a significant decrease in total GSH levels and increase in oxidized glutathione disulfide (GSSG), with a consequent deficit in the GSH/GSSG redox ratio and GSH-mediated antioxidant capacity. These effects are associated with increased *S*-glutathionylation of distinct proteins involved in adaptive responses to oxidative stress, including redox-sensitive chaperonins, metabolic enzymes, and cytoskeletal proteins, suggesting a novel molecular signature of KRIT1 loss-of-function that could contribute to its emerging pleiotropic effects in the pathogenesis of CCM disease.

Taken together, our findings suggest that changes in the GSH/GSSG redox potential associated with KRIT1 loss-of-function result in enhanced *S*-glutathionylation of distinct regulatory and structural proteins, which can in turn contribute to the emerging redox-sensitive pleiotropic functions of KRIT1, thus defining a novel molecular signature as well as pointing to the potential identification of new disease biomarkers and therapeutic targets (Figure 9). Consistently, as an important mechanism for dynamic and coordinated post-translational regulation of a variety of regulatory, structural, and metabolic proteins, PSSG is ideally suited for integrating and mounting fine-tuned responses to changes in the redox state in both physiological and pathological conditions, and has already suggested useful translational implications [16,17,44,92]. In particular, the assessment of *S*-glutathionylated blood proteins has gained a diagnostic/prognostic value, serving as a possible indicator of oxidative stress in correlation with the evolution of some human diseases [46,93,94] and could be therefore considered in the context of CCM disease. Furthermore, given the established role of PSSG as a critical signaling mechanism in cardiovascular health and disease [82], and considering the strong evidence that oxidative stress can alter the activity of major proteins involved in fundamental endothelial cell functions through PSSG [75], it is also possible to speculate that perturbations in PSSG status may contribute to the emerging role of oxidative stress in the pathogenesis of CCM disease, thus paving the way for a novel research avenue. 

Future studies aimed at an extended coverage of *S*-glutathionylated proteins associated with KRIT1 loss-of-function, including the use of a gel-free approach and a deep characterization of their functional implications, should provide useful insights for a better understanding of CCM disease pathogenesis, and new options for the identification of useful biomarkers and the development of targeted, safe and effective therapeutic strategies, which could benefit from the features of pleiotropy and reversibility of the PSSG process.

## Figures and Tables

**Table 1:** Mass spectrometry analysis of tryptic digests from *S*-glutathionylated proteins. Spot number, SwissProt accession, protein description, gene name, Mascot score, number of unique peptides identified, sequence coverage, and theoretical mass and pI values are reported.

Spot	SwissProt Accession	Protein Description	Gene Name	Mascot Score	Unique Peptides	Sequence Coverage (%)	Mass (Da)	pI
1	P16858	Glyceraldehyde-3-phosphate dehydrogenase	Gapdh	165	3	7.8	36,072	8.44
2	P16858	Glyceraldehyde-3-phosphate dehydrogenase	Gapdh	95	2	7.5	36,072	8.44
3	P16858	Glyceraldehyde-3-phosphate dehydrogenase	Gapdh	100	2	7.8	36,072	8.44
4	P17182	Alpha-enolase	Eno1	553	12	29.7	47,453	6.37
5	P17182	Alpha-enolase	Eno1	818	15	42.2	47,453	6.37
6	P17182	Alpha-enolase	Eno1	710	14	39.9	47,453	6.37
7	P63038	60 kDa heat shock protein	Hspd1	856	14	34.2	61,088	5.91
8	P20152	Vimentin	Vim	993	22	50.0	53,712	5.06
9	P20152	Vimentin	Vim	968	21	50.4	53,712	5.06
10	P14211	Calreticulin 1	Calr	163	4	8.4	48136	4.33
11	Q04447	Creatine kinase B-type	Ckb	190	4	16.5	42,971	5.4
12	Q04447	Creatine kinase B-type	Ckb	179	4	16.8	42,971	5.4
13	P68372	Tubulin beta-4B chain	Tubb4b	582	11	29.4	50,255	4.79
14	P60710	Actin, cytoplasmic 1	Actb	185	4	17.3	42,052	5.29
15	P60710	Actin, cytoplasmic 1	Actb	341	7	22.7	42,052	5.29
22	P20152	Vimentin	Vim	945	22	43.8	53,712	5.06
23	P20152	Vimentin	Vim	961	20	47.9	53,712	5.06
24	P60710	Actin, cytoplasmic 1	Actb	276	5	16.3	42,052	5.29
25	P60710	Actin, cytoplasmic 1	Actb	276	5	16.3	42,052	5.29
26	Q58E70	Tpm 3 protein	Tpm3	502	10	37.9	29,231	4.75
	Q6IRU2	Tropomyosin alpha-4 chain	Tpm4	308	6	27.0	28,564	4.65
